# Designing an Additively Manufactured Ti-Al-Fe Alloy with a Wide Process Window

**DOI:** 10.3390/ma18214986

**Published:** 2025-10-31

**Authors:** Leyu Cai, Zixuan Hong, Feng Xu, Xinyan Liu, Ziyuan Zhao, Jing Peng, Qihong Fang, Hong Wu

**Affiliations:** 1 State Key Laboratory of Powder Metallurgy, Central South University, Changsha 410083, China; asxx41239@126.com (L.C.); hhongzixuan@163.com (Z.H.); xufeng.am@gmail.com (F.X.); liuxinyan@farsoon.com (X.L.); zziy916@163.com (Z.Z.); 2 Farsoon Technologies Co., Ltd., Changsha 410221, China; 3 State Key Laboratory of Advanced Design and Manufacturing for Vehicle Body, College of Mechanical and Vehicle Engineering, Hunan University, Changsha 410082, China; jingpeng@hnu.edu.cn (J.P.); fangqh1327@hnu.edu.cn (Q.F.)

**Keywords:** titanium alloy, laser powder bed fusion, process window, microstructure, mechanical property

## Abstract

To develop a cost-effective titanium alloy tailored for laser powder bed fusion (LPBF), a novel Ti-5.2Al-5Fe (wt.%) dual-phase alloy was designed and fabricated in this study. The composition was optimized for low density (4.4 g/cm^3^), high yield strength (1052 MPa), and suitable β-phase stability ([Mo]_eq_ = 9.3%). The alloy demonstrated excellent formability, achieving high densification (porosity ≤ 2%) and hardness (>400 HV) over a wide volumetric energy density range (48–204 J/mm^3^). The Al element inhibited balling by improving melt pool wettability, while the Fe element synergistically promoted densification by lowering the liquidus temperature. The as-built microstructure comprised α and β phases, with the α-phase content increasing significantly from 25.4% to 60.8% with higher energy density. While all samples exhibited high tensile strength (>1290 MPa), ductility was limited (<2.6%). EBSD analysis identified the α-phase as the primary carrier of micro-residual stress, with a high density of “zero-solution” points, low-angle grain boundaries, and KAM values. This indicates severe stress concentration from rapid solidification and phase transformation, elucidating the fundamental reason for the low ductility. This study provides systematic insights from composition design to microscopic mechanisms for designing LPBF-dedicated titanium alloys with a wide process window.

## 1. Introduction

Additive manufacturing (AM) technologies, such as laser powder bed fusion (LPBF), demonstrate significant advantages in fabricating components with complex geometries, enabling lightweight design and functional integration, thereby offering innovative solutions for fields like aerospace and biomedical engineering [1,2]. Titanium alloys, owing to their excellent specific strength, corrosion resistance, and biocompatibility, have become a key material system for this technology [3]. However, the titanium alloy compositions widely used in LPBF (e.g., Ti-6Al-4V) were primarily designed to meet the requirements of traditional casting or forging processes. These conventional alloy systems struggle to fully adapt to the inherent characteristics of LPBF, such as rapid melting and solidification as well as non-equilibrium thermal cycling. These characteristics often lead to a narrow process window, defect formation, or the development of coarse columnar grains [4,5,6]. Numerous studies have achieved successful additive manufacturing of these compositions through parameter optimization, substrate preheating, or the addition of grain refiners [7,8,9,10]. Nevertheless, these titanium alloy systems fail to fully exploit the potential advantages of “non-equilibrium metallurgy”, consequently limiting further enhancement of component performance.

From a materials development perspective, the LPBF process itself can be viewed as a unique in situ alloying process. The inherent rapid melting and solidification of metal powders, coupled with ultrafast cooling rates as high as 10^3^–10^8^ K/s, provides researchers with an in situ alloying environment dominated by unique melt pool dynamics [11,12,13]. This environment makes it possible to directly form metastable phases and specific microstructures through rapid solidification, thereby opening new pathways for optimizing alloy properties [14]. Precisely for this reason, the research frontier is gradually shifting from passively “making the process adapt to the material” to proactively “designing the material for the process”, aiming to develop new alloy compositions specifically tailored to the characteristics of LPBF to fully unlock the technology’s potential in alloy design and mechanical performance enhancement.

Against this backdrop, this study is dedicated to designing a novel titanium alloy suitable for LPBF, with a design philosophy closely aligned with the industrial need for efficient resource utilization. Over 90% of titanium resources in nature exist in the form of ilmenite, characterized by high iron content [15]. In contrast, within current titanium alloy systems, iron is regarded as an impurity that requires strict control. Its deep removal process is costly and generates large amounts of secondary sponge titanium scrap that cannot be utilized due to excessive iron and oxygen content [16]. Therefore, developing new titanium alloy systems capable of tolerating and actively utilizing high iron content is a crucial path to breaking the resource bottleneck and reducing overall costs. Based on this context, this study aims to design a high-iron titanium alloy that adapts to these resource characteristics. Considering that α + β dual-phase titanium alloys offer a good balance of strength, toughness, and hot workability, they represent the ideal target for this design [17,18,19]. Consequently, we selected the most common and low-cost α-stabilizing element Al [20] and the β-stabilizing element Fe [21] as the primary alloying components, exploring the potential of utilizing this Ti-Al-Fe system to achieve a wide process window and good mechanical properties in the LPBF process.

Therefore, the objective of this study is to verify whether, through composition design based on the Ti-Al-Fe system, a titanium alloy with adaptability to the additive manufacturing process can be obtained without relying on external aids such as substrate preheating. This study systematically investigates the densification behavior, process window, microstructure, and mechanical properties of the designed Ti-5.2Al-5Fe (wt.%) alloy during the LPBF process. The findings are expected to provide new design insights for developing low-cost, highly adaptable, high-performance titanium alloys dedicated to LPBF.

## 2. Experimental

### 2.1. Composition Design

**Figure 1:**
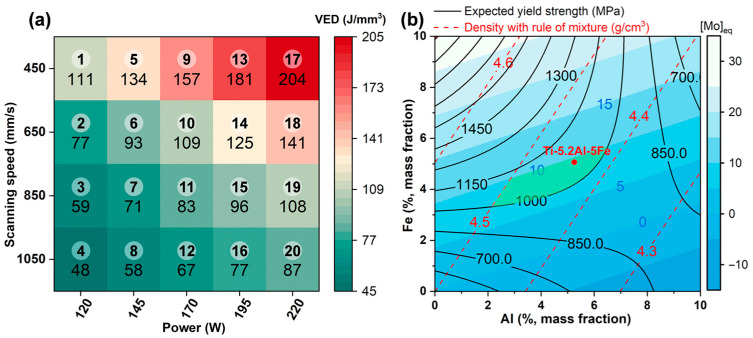
Composition design and process parameter selection for the Ti-Al-Fe alloy: (**a**) applied process parameters and corresponding sample designations; (**b**) composition design range determined based on density minimization, yield strength optimization, and β-phase stability criteria.

Al, as an α-phase stabilizer, provides matrix strengthening and high-temperature stability [22,23]. Fe, as a β-phase stabilizer, is the most potent and economical β-stabilizing element known [24]. Furthermore, both Al and Fe have low atomic masses, which helps maintain the lightweight advantage of titanium alloys. Additionally, significant differences in the diffusion rates of Fe and Al elements in β-Ti and α-Ti were key reasons for selecting Al and Fe as solute elements [25]. Based on current research, and aiming to obtain a dual-phase titanium alloy with low density and good mechanical properties, this study followed three design criteria:

(i) Density minimization. The composition was selected based on the rule of mixtures [26] to achieve a theoretical density lower than that of pure Ti. In the contour map used for composition screening (Figure 1b), the red contour lines represent the theoretical density calculated according to the rule of mixtures.
(ii) Yield strength optimization. Solid-solution strengthening is a core mechanism determining yield strength. This study utilized the yield strength prediction model proposed by Liang et al. [27]. Due to insufficient mechanical test data for Ti-Al-Fe alloys fabricated by additive manufacturing, the model used yield strength data from cast-formed alloys, which is a net-shape manufacturing process similar to AM. This model comprehensively considers the contributions of individual elements and their interactions, expressed as: $\sigma_{YS}=\sum \sigma_{M}X_{M}+\sum L_{M-N}X_{M}X_{N}$, where M and N represent the elements in the alloy, $\sigma_{M}$ represents the yield strength of the corresponding pure metal, $X_{M}$ represents the mole fraction of the element in the alloy, and $L_{M-N}$ represents the interaction parameter between the two elements in a binary alloy. Alloy compositions with a predicted yield strength greater than 1000 MPa were selected. In the composition diagram, the black contour lines intuitively illustrate the distribution of yield strength predicted based on this solid-solution-strengthening model.
(iii) β-phase stability. To obtain a dual-phase titanium alloy, compositions with a Mo equivalent [Mo]_eq_ slightly below the lower stability limit for the β phase (10 wt.%) [28] were selected. The varying shades of blue background in the composition diagram directly correspond to the values of the molybdenum equivalent, which is used to evaluate the stability of the β phase.

Using Origin software (version 2022), the contour maps corresponding to these three design criteria were overlaid into a single composition diagram, as shown in Figure 1b. This allows for intuitive and convenient identification of the composition range that simultaneously meets the requirements of low density, high predicted strength, and appropriate β-phase stability—specifically, the green region indicated in the figure. Based on this, the composition Ti-5.2Al-5Fe (wt.%) was selected for this study. This composition corresponds to a theoretical density of 4.4 g/cm^3^, a predicted yield strength of 1052 MPa, and a [Mo]_eq_ of 9.3 wt.%.

### 2.2. Materials Processing

**Figure 2:**
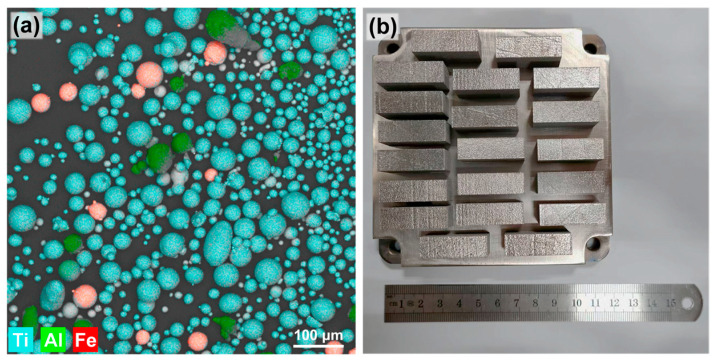
(**a**) SEM morphology and elemental distribution of the mixed powders; (**b**) macrograph of the as-built Ti-Al-Fe alloy specimens.

Elemental powders of spherical gas-atomized pure Ti, pure Al, and pure Fe powders (purity 99.9%), purchased from Beijing Kairui New Material Co., Ltd. (Beijing, China), were blended to prepare the alloy, rather than using a pre-alloyed powder. The elemental powders were mixed in a mass ratio of Ti:Al:Fe = 89.8:5.2:5 using a powder mixer for 24 h. As shown in Figure 2a, no alloying occurred between the powders after mixing.

The LPBF process was carried out using an FS 121 M metal 3D printer, purchased from Hunan Farsoon High-Technology Co., Ltd. (Changsha, China). The morphology of the as-deposited part is shown in Figure 2b. During the LPBF process, a laser spot diameter of 60 μm, a powder layer thickness of 30 μm and a hatch spacing of 80 μm were used. Laser powers of 120, 145, 170, 195, and 220 W were applied in combination with scan speeds of 450, 650, 850, and 1050 mm/min. The corresponding volumetric energy density (VED) was calculated to range from 48 to 204 J/mm^3^. The respective samples were designated S1 through S20, as shown in Figure 1a. A pure Ti substrate was used, and argon was employed as the protective atmosphere. The scanning strategy involved a 90° rotation between subsequent layers. The oxygen and nitrogen contents of the as-built specimens are provided in Table S1.

### 2.3. Microstructural Characterization

The as-built parts were sectioned perpendicular to the build direction (BD) to obtain bulk specimens and tensile specimens. The specific dimensions and cutting locations are detailed in Figure S1. The phase composition of the powders and as-deposited parts was characterized using a Bruker Advance D8 X-ray diffractometer (XRD; Karlsruhe, Germany) with Cu-*K*_α_ radiation. XRD scans were performed with a step size of 0.02° and a scan speed of 0.2 s/step over a 2*θ* range of 20° to 90°. Subsequently, the sample surfaces were polished to a mirror finish for observation using a Leica DM2700P optical microscope (OM; Wetzlar, Germany) and a Regulus 8230 cold-field emission scanning electron microscope (SEM; Tokyo, Japan). For electron backscatter diffraction (EBSD) characterization (using a JSM-7200F, JEOL; Tokyo, Japan), the samples were further prepared by electrolytic polishing. The EBSD scans were conducted with a step size of 0.7 μm at an accelerating voltage of 20 kV.

### 2.4. Porosity Analysis

The porosity of the material was quantitatively analyzed using the metallographic method. Optical microscopy was performed at 200× magnification to capture images from 15 randomly selected fields of view on the polished sample surface. The pore areas (dark pixels) were segmented from the matrix areas (bright pixels) via binarization processing using ImageJ software (version 1.53q), allowing for the measurement of both the pore area and the total inspected area. The porosity was determined by calculating the ratio of the total pore area to the corresponding total inspected area in each field of view, with the final result reported as the average of these measurements.

### 2.5. Mechanical Testing

Microhardness was measured using a Buehler-5104 microhardness tester (Buehler Ltd., Lake Bluff, IL, USA) with a load of 200 g and a dwell time of 15 s. At least five indentations were made on the surface of each sample. Quasi-static, room-temperature uniaxial tensile tests were conducted in accordance with the GB/T 228.1-2021 standard [29], using an AGS-X electronic universal testing machine (Shimadzu Corporation; Kyoto, Japan). The tests were performed at a constant engineering strain rate of 10^−3^ s^−1^, with each group of specimens tested repeatedly for 4 times.

## 3. Results and Discussion

### 3.1. Formability of LPBF-Fabricated Ti-Al-Fe Alloy

**Figure 3:**
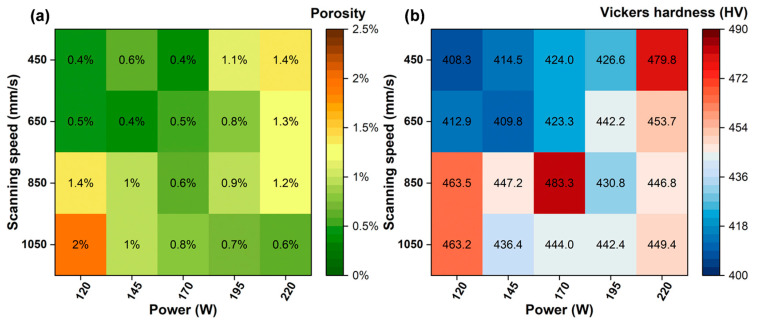
Influence of process parameters on formability and hardness: (**a**) porosity; (**b**) Vickers hardness.

**Figure 4:**
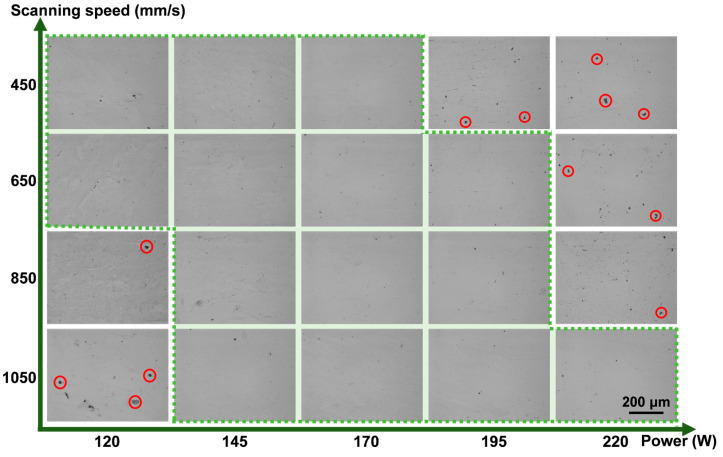
Pore size and distribution obtained from metallographic image analysis.

Figure 3 shows the porosity and hardness of the Ti-5.2Al-5Fe alloy fabricated under different processing parameters. Within the parameter range investigated in this study, all samples exhibited porosity ≤ 2% and hardness > 400 HV. As observed from the metallographic images (Figure 4) showing the pore size and distribution in alloys formed under different parameters, the sample porosity could be maintained at ≤1% even across a relatively wide volumetric energy density range of 60–157 J/mm^3^ (delineated by the green dashed frame), demonstrating favorable formability and densification. Observation of the specimens revealed two distinct types of pores with markedly different morphologies and sizes: one type consisted of larger pores (red circles in Figure 4) with an average diameter of approximately 16.8 μm, often exhibiting irregular contours; the other type comprised fine pores with an average diameter of about 0.8 μm, predominantly displaying regular circular shapes, which were present in most specimens. Based on their size and morphological characteristics, the pores can be classified into two types: the large pores are keyhole pores, and the small pores are gas pores. Gas pores are one of the most common defects in laser additive manufacturing, primarily related to powder characteristics and process instability [4]. The formation of keyhole pore defects, conversely, is closely related to the violent vaporization of material under the high-energy beam [5].

**Figure 5:**
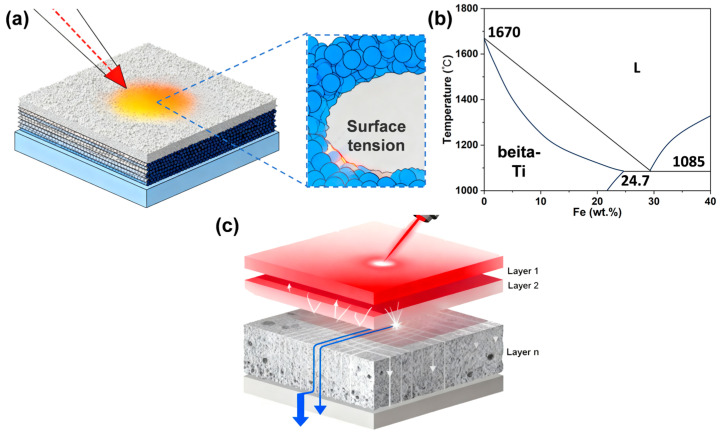
Mechanisms for the good LPBF formability of the alloy: (**a**) reduced melt surface tension by Al; (**b**) lowered liquidus temperature through the use of Fe (illustrated using the Ti-Fe phase diagram [33]); (**c**) schematic of the layer-by-layer fabrication process in LPBF.

The Ti-5.2Al-5Fe alloy demonstrated an exceptionally wide process window during LPBF. The Al element was crucial for improving the wettability and fluidity [22]. As shown in Figure 5a, during rapid laser melting, the alloy powder is rapidly molten to form a melt pool. Due to its relatively low surface tension and favorable intermiscibility for the titanium melt, Al effectively reduces the overall surface tension of the melt and significantly improves the wettability between the liquid titanium alloy and the previously printed layer beneath [30,31]. This excellent wettability ensures the melt pool flattens and spreads uniformly, avoiding the “balling” effect, which is fundamental for forming continuous, dense melt tracks. Fe, as a strong β-stabilizer, primarily influences the process by significantly lowering the liquidus temperature of the alloy (Figure 5b) and substantially narrowing the solidification temperature range [32,33]. A narrow solidification range is crucial for additive manufacturing because it means the time from the start of crystallization to complete solidification of the melt pool is very short. This confers two key advantages: First, it greatly suppresses dendritic growth and solute segregation. Under rapid cooling, Fe is more effectively retained within the β-phase grains, forming a supersaturated solid solution and reducing the risk of forming low-melting-point brittle phases in interdendritic regions due to severe segregation, which are often the initiation sites for solidification cracks. Second, the narrow solidification range reduces the viscosity of the melt pool in the final stages of solidification, enhancing the liquid feeding to compensate for shrinkage more effectively. Thereby reducing the tendency for hot cracking and the formation of micro-porosity induced by solidification shrinkage. Therefore, by modifying the solidification kinetics itself, Fe creates thermodynamically and kinetically favorable conditions for obtaining a dense, defect-free deposited microstructure. To further investigate the optimized solidification behavior and elemental distribution achieved by the combined effects of Al and Fe, SEM micrographs and corresponding elemental mapping of the as-built samples were analyzed, as shown in Figure S2. Under the optimized processing parameters, the deposited samples exhibited a dense and homogeneous microstructure. Notably, with increasing volumetric energy density, the distribution of Al and Fe elements became progressively more uniform at the microscale, directly confirming the effectiveness of rapid solidification in suppressing solute segregation.

Under conditions where both the scan speed and laser power were low, the alloy exhibited its lowest hardness (Figure 3b). This is attributed to the relatively low peak temperature of the single melt pool caused under the low laser energy input, resulting in a smaller melt pool volume, shorter existence time, and a relatively slower cooling rate. As shown in Figure 5c, additive manufacturing is a layer-by-layer process. At lower scan speeds, the laser dwells longer on each specific area. Consequently, when subsequent layers are printed, the previously solidified layers undergo longer, slower heating and cooling cycles—essentially multiple, prolonged thermal cycles [34]. The peak temperatures of these repeated thermal effects, while potentially below the solidus line, are sufficient to reach medium-to-high temperature ranges, thereby imparting an in situ annealing effect on the material, resembling step-wise or sustained annealing [35]. These repeated thermal cycles provide sufficient thermal activation for atomic diffusion, facilitating the rearrangement, annihilation, or formation of low-energy dislocation cell structures from the high-density dislocations generated during rapid solidification. This effectively releases residual stresses, directly leading to a significant reduction in the macro-hardness of the material [36,37,38].

Hardness testing provides a rapid and cost-effective method for localized property assessment. The relationship between the strength and hardness of LPBF titanium alloys [39] can be utilized to predict the yield strength of components: $\sigma_{0.2}\left(MPa\right)=\frac{HV\left(MPa\right)}{3.60}-90$, where *HV* represents Vickers hardness and requires unit conversion. While this indirect method for strength prediction is efficient, its accuracy may be influenced by microstructural variations. Accordingly, three samples (S4, S11, S17) which exhibited high hardness despite having different VEDs were selected for further analysis.

### 3.2. Tensile Properties of LPBF-Fabricated Ti-Al-Fe Alloy

**Figure 6:**
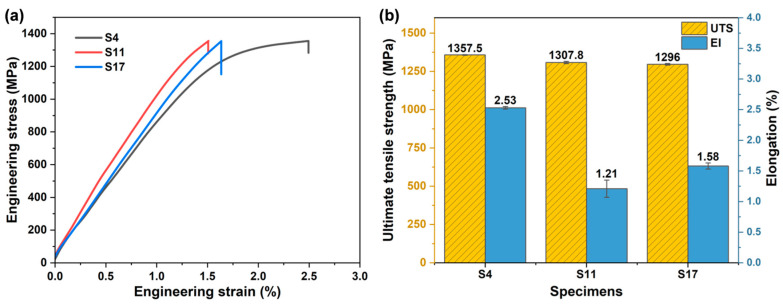
Effect of process parameters on room-temperature tensile properties: (**a**) tensile engineering stress–strain curves; (**b**) ultimate tensile strength and elongation.

Figure 6a shows the room-temperature tensile engineering stress–strain curves for S4, S11, and S17. The results indicate that the process parameters had a minor influence on the strength of the as-deposited parts. As shown in Figure 6b, the yield strength σ_0.2_ of S4 was 1231.9 MPa. The ultimate tensile strengths (UTS) for the three samples were 1357.5 ± 2.5 MPa, 1307.8 ± 7.1 MPa, and 1296.0 ± 5.6 MPa, respectively, with elongations of 2.53 ± 0.02%, 1.21 ± 0.14%, and 1.58 ± 0.05%, respectively. Within the process parameter window of this study, variations in laser power and scan speed did not significantly affect the room-temperature tensile strength of the as-deposited titanium alloy. High residual tensile stress is a primary cause of cracking and premature plastic failure in AM components [40]. The multiple, slow thermal cycles experienced by S4 significantly relaxed the internal residual stresses and avoided stress concentration, enabling the material to undergo more uniform plastic deformation during tension rather than premature fracture. However, S4, fabricated with high scan speed and low laser power, exhibited better elongation, which may be related to its phase composition and grain size. Furthermore, tensile specimens in this study were all extracted from the same height across different samples. The potential variations in mechanical properties at different heights within a component have not been thoroughly investigated, which is an essential consideration before applying this alloy to actual complex components.

**Figure 7:**
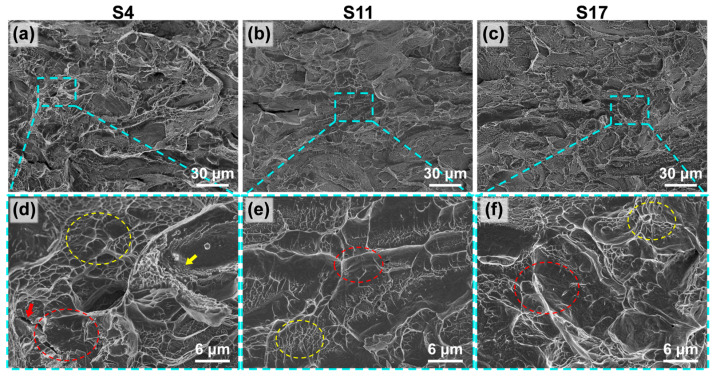
Fracture surface morphology of samples fabricated at VEDs: (**a**–**c**) macroscopic views; (**d**–**f**) higher magnification views of the regions indicated by the blue squares in (**a**–**c**).

**Figure 8:**
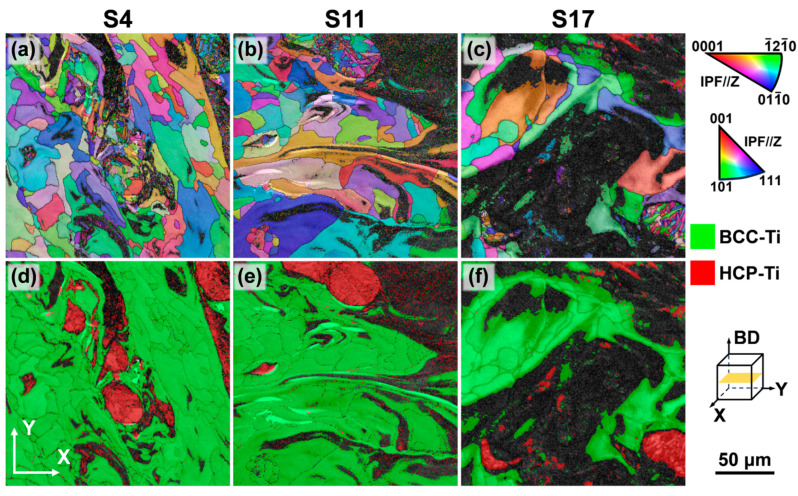
EBSD analysis of Ti-5.2Al-5Fe fabricated at different VEDs: (**a**–**c**) IPF maps; (**d**–**f**) phase distribution maps.

Figure 7 shows the morphology of the cross-sections beneath the tensile fracture surfaces for samples fabricated at different energy densities. The fracture surfaces of all samples exhibited two distinct types of regions: one region uniformly distributed with dimples (red circles), indicative of the ductile fracture zone; and another region that was smooth and flat (yellow circles), corresponding to the brittle fracture zone. Combined with the EBSD analysis results shown in Figure 8, all three samples consisted of both α-Ti (HCP structure) and β-Ti (BCC structure) phases. The α-Ti phase has higher strength but poorer ductility [41], and its regions primarily exhibit characteristics of brittle fracture on the fracture surface. In contrast, the β-Ti phase exhibits greater ductility [42], corresponding to the dimpled regions, i.e., the ductile zones. As the energy density increased, the area of the brittle zone on the fracture surface gradually expanded. This expansion of the brittle zone is consistent with the increased content of the α phase, indicating that higher energy density input promotes the formation or retention of the α phase, thereby increasing the propensity for brittle fracture. Furthermore, due to the high residual tensile stress accumulated during the additive manufacturing process [6], the material could not undergo sufficient plastic deformation before fracture. This resulted in relatively shallow dimples in all samples, reflecting their limited local ductility. The average dimple diameters for samples S4, S11, and S17 were 1.1, 0.5, and 0.4 μm, respectively, which gradually decreased with increasing energy density. Notably, on the fracture surface of sample S4, which had higher elongation, micro-cracks (red arrows) and a small amount of unmelted/unfused powder (yellow arrows) were observed in addition to dimples. These features are attributed to its fabrication conditions of low laser power and high scan speed. Such defects can act as stress concentration sources during tensile testing.

### 3.3. Effect of Volumetric Energy Density on Microstructure

**Figure 9:**
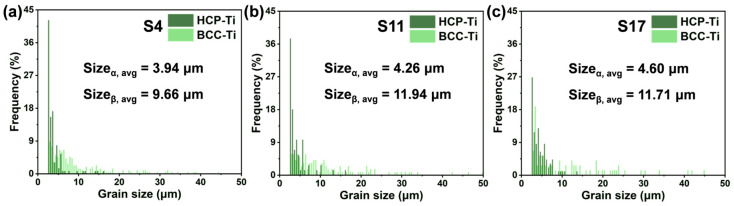
Grain size distribution of the Ti-5.2Al-5Fe fabricated at different VEDs: (**a**) S4; (**b**) S11; (**c**) S17.

Figure 8a–c present the inverse pole figure (IPF) maps of samples fabricated at different VEDs, illustrating a heterogeneous microstructure composed of coarse and fine grains. By comparing these with the phase distribution maps of the same regions (Figure 8d–f), it is evident that the coarse-grained regions primarily correspond to the β phase, while the fine-grained regions are correlated with the α phase. Grain size statistics based on the equivalent circle diameter, shown in Figure 9, further confirm that the α grains are significantly finer than the β grains. This is primarily attributed to the α phase being a secondary phase that transforms from the high-temperature β phase upon cooling. Its high nucleation rate and confined growth space at prior β grain boundaries and within the grains typically result in the formation of fine lamellar or equiaxed structures [43]. As the VED increases, the grain size of both α and β phases exhibits coarsening. This occurs because higher energy input (higher laser power or lower scan speed) results in a slower cooling rate, providing a longer thermal duration for grain growth. This heterogeneous structure combining coarse and fine grains can potentially enhance strength by introducing heterogeneous plastic deformation capability [14]. However, all samples in this study exhibited limited elongation, indicating that the detrimental effect of high residual stress accumulated during the additive manufacturing process on plasticity dominates.

**Figure 10:**
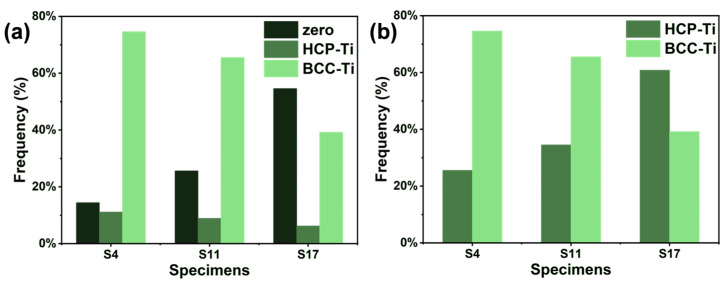
Phase fractions of the Ti-5.2Al-5Fe fabricated at different VEDs: (**a**) as-measured from EBSD; (**b**) reconstructed after incorporating zero-solution areas.

Analysis of the phase area fractions (Figure 10) revealed that as VED increases, the fraction of “zero-solution” points in the EBSD analysis increases significantly. This phenomenon is caused by significant residual stress concentration within the α phase regions, which degrades or distorts the Kikuchi diffraction patterns, making reliable phase indexing difficult [44]. The concentration of residual stress in the α phase stems mainly from the volumetric change accompanying the β→α solid-state phase transformation. Under the rapid, non-equilibrium cooling conditions of LPBF, this transformation strain cannot relax sufficiently, becoming stored in the α phase as lattice distortion and micro-stresses [45]. In contrast, the β phase, as the high-temperature parent phase, is inherently more ductile, and martensitic transformation during cooling is suppressed, resulting in relatively lower residual stress levels. Based on this, it can be reasonably inferred that these “zero-solution” regions are essentially α phase areas with high stress concentration. By adding the area of the zero-solution regions to the identified α phase area, the actual α phase area fractions for samples S4, S11, and S17 are calculated to be 25.4%, 34.5%, and 60.8%, respectively. This demonstrates that the α phase content in the dual-phase titanium alloy can be effectively controlled over a wide range by adjusting the LPBF process parameters. The significant increase in α phase content with higher VED is attributed to the fact that the prolonged melt pool existence time and reduced cooling rate under high VED conditions provide greater thermodynamic driving force and longer diffusion time for the β→α solid-state phase transformation, promoting the growth of more α phase. Additionally, substrate preheating is an effective method for controlling residual stress in LPBF. It was not employed in this study, however, in order to focus on the inherent process adaptability of the alloy composition itself.

**Figure 11:**
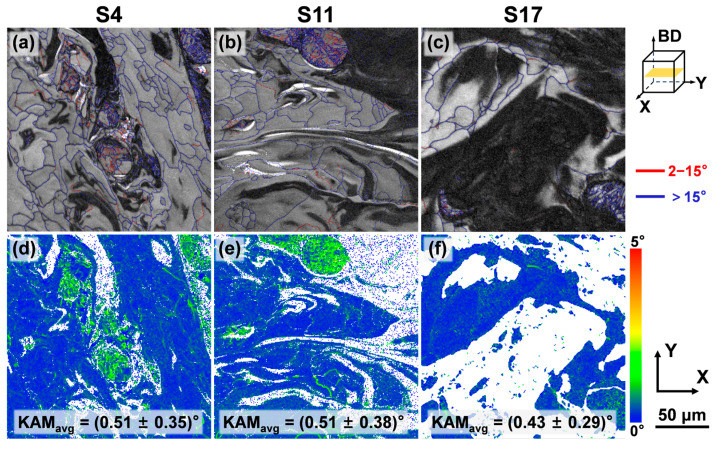
Microstructural characteristics of Ti-5.2Al-5Fe fabricated at different VEDs: (**a**–**c**) distributions of HAGBs and LAGBs; (**d**–**f**) KAM maps and the corresponding average KAM values.

Low-angle grain boundaries (LAGBs), defined by misorientations between 2° and 15°, are closely associated with dislocation slip and reorganization, and their density directly reflects the magnitude of plastic strain and dislocation density within the material [46]. From the grain boundary distribution maps shown in Figure 11a–c, it is apparent that a high density of LAGBs is distributed within and at the boundaries of the α phase, with a density significantly higher than that in the β phase. The boundaries of the β grains, conversely, consist predominantly of high-angle grain boundaries (HAGBs). The specific fractions of LAGBs for each sample are listed in Table 1. The high density of LAGBs in the α phase is primarily attributed to its HCP crystal structure, which has fewer slip systems and poorer plastic deformation compatibility. Under the influence of rapid cooling and associated thermal stresses, dislocation pile-ups readily form LAGBs to accommodate the transformation strain and thermal stress [41].

The Kernel Average Misorientation (KAM), derived from EBSD data, is commonly used to indicate grain-scale plastic deformation [47]. Micro-strain helps maintain lower strain values by restricting the slip of the geometrically necessary dislocations (GNDs). The presence of micro-strain facilitates the transformation between GNDs and LAGBs. Dislocations, as carriers of plasticity, contribute to increased micro residual stress, i.e., local lattice distortion, which ultimately leads to residual stress at the macro level. Residual stress is partially or fully relieved through the reduction in dislocation density and twins, which is reflected in a decrease in the mean KAM value. The lattice strain and macro-strain induced by a certain stress state are consistent [46]. Higher macro-stress results in more pronounced differences in interplanar spacing between grains of different orientations, and larger misorientation angles between grains. Since a suitable method has not been established to accurately and directly measure the residual stress distribution across the entire plane of the sample, we opted to use KAM to investigate the effect of residual stress. As shown in Figure 11d–f, the KAM values in the α-phase regions corresponding to the α phase are significantly higher than those in the β phase. Additionally, high KAM values are also observed at the boundaries of some β grains. This result clearly indicates that the α phase is the primary carrier of micro-scale residual stress and lattice distortion in this alloy system, consistent with the observation of high zero-solution areas in EBSD. These high KAM regions represent potential sites for crack initiation and propagation, explaining the limited macroscopic plasticity.

## 4. Conclusions

This study successfully fabricated a dual-phase Ti-5.2Al-5Fe (wt.%) titanium alloy via LPBF, systematically investigating the effects of process parameters on its formability, microstructure, and mechanical properties. Main conclusions are as follows:

(1) The Ti-5.2Al-5Fe alloy, designed based on criteria of density, strength, and β-phase stability, achieved high densification (porosity generally ≤ 2%) across a wide energy density range of 48–204 J/mm^3^. The synergistic effects of Al (improving melt pool wettability) and Fe (lowering the liquidus temperature) collaboratively broadened the process window.
(2) The as-deposited microstructure consisted of α + β dual phases, with the α-phase content increasing from 25.4% to 60.8% as the energy density rose. EBSD analysis revealed that the α-phase acts as the primary carrier of micro-residual stress, as evidenced by a high fraction of “zero-solution” points, a high density of low-angle grain boundaries, and elevated KAM values, all indicating significant lattice distortion within the α-phase.
(3) The alloy exhibited high strength but low ductility in the as-deposited state, with tensile strength exceeding 1290 MPa but elongation generally below 2.6%. The high strength originated from heterogeneous structures and solid-solution strengthening, while the limited ductility was primarily attributed to residual stress concentration in the α-phase regions.

This study clarified the ductility limitations and stress distribution in the as-built alloy. However, systematic heat treatment studies and validation of its applicability to complex components are still lacking. Future work will focus on regulating stress and phase composition via heat treatment, and evaluating the formability and performance consistency of practical components.

## Figures and Tables

**Table 1:** Quantitative EBSD analysis of the Ti-5.2Al-5Fe fabricated at different VEDs.

Parameters	S4			S11			S17		
	α	β	Entirety	α	β	Entirety	α	β	Entirety
Fraction of area (%)	25.4	74.6	-	34.5	65.5	-	60.8	39.2	-
Average grain size (μm)	3.94	9.66	-	4.26	11.94	-	4.60	11.71	-
Fraction of LAGBs (%)	-	-	14.4	-	-	16.0	-	-	7.5
KAM value (°)	0.80	0.46	0.51	0.78	0.48	0.51	0.57	0.42	0.43

## Data Availability

The raw data supporting the conclusions of this article will be made available by the authors on request.

## Supplementary Materials

The following supporting information can be downloaded at: https://www.mdpi.com/article/10.3390/ma18214986/s1, Figure S1: Supplementary explanation for tensile test: (**a**) schematic diagram of sample cutting method and (**b**) schematic diagram of tensile specimen size and morphology; Figure S2: SEM micrographs of the as-printed specimens and corresponding EDS mapping analysis results; Table S1: O and N contents (wt.%) in the as-printed Ti-5.2Al-5Fe alloy.
