# Supplementary material for: Designing an Additively Manufactured Ti-Al-Fe Alloy with a Wide Process Window
Source: Materials (Basel). 2025 Oct 31;18(21):4986. doi: 10.3390/ma18214986 (PMC12608022; doi:10.3390/ma18214986)

Supplementary materials for design of an additively manufactured Ti-Al-Fe alloy with a wide process window

Supplementary Table S1. O and N contents (wt.%) in the as-printed Ti-5.2Al-5Fe alloy. Interstitial solid solution elements such as O and N have a significant impact on the ductility of as-printed specimens. These elements have a very low content threshold in the alloy; once this upper limit (0.2 wt.%) is exceeded, the ductility decreases rapidly. The laser powder bed fusion equipment used in this work adopts an integral sealed structure. During the preparation process, Ar gas is introduced as a protective gas, and a gas circulation purification system is configured. This strictly controls the oxygen content below 50 ppm and the water content below 50 ppm in the atmosphere protection chamber, effectively preventing severe oxidation of the metal. However, we consider that the elemental powders used for in-situ alloying may also lead to the introduction of O or other impurity elements. Therefore, in accordance with the GB/T 14265-2017 standard, we tested the O and N contents in the three specimens using the reduction fusion method, with each group of tests repeated 3 times. By comparing with the chemical compositions of titanium alloys listed in GB/T 3620.1-2016, it can be concluded that the O/N contents in the Ti-5.2Al-5Fe prepared in this study are controlled within a reasonable range and are not the main cause of such low plasticity.

element	S4	S11	S17
O	0.093 ± 0.002	0.107 ± 0.006	0.102 ± 0.005
N	0.046 ± 0.003	0.034 ± 0.002	0.037 ± 0.005

Supplementary Figure S1. Supplementary explanation for tensile test: (a) schematic diagram of sample cutting method and (b) schematic diagram of tensile specimen size and morphology. The dimensions of the tensile specimens in this experiment were determined in accordance with GB/T 228.1-2021. To eliminate the influence of the height of as-printed specimens on mechanical properties, we ensured all specimens had the same height and obtained tensile specimens exclusively from the limited range at the mid-height of the as-printed specimens. Additionally, the yield strength mentioned in the article is $\sigma_{0.2}$. $\sigma_{0.2}$ refers to the strength corresponding to the intersection of the offset line and the 0-stress axis is at 0.2% strain. This offset yield strength is determined from a stress-strain curve. For each group, we derived $\sigma_{0.2}$ from the stress-strain curve of each individual specimen, calculated the average value.

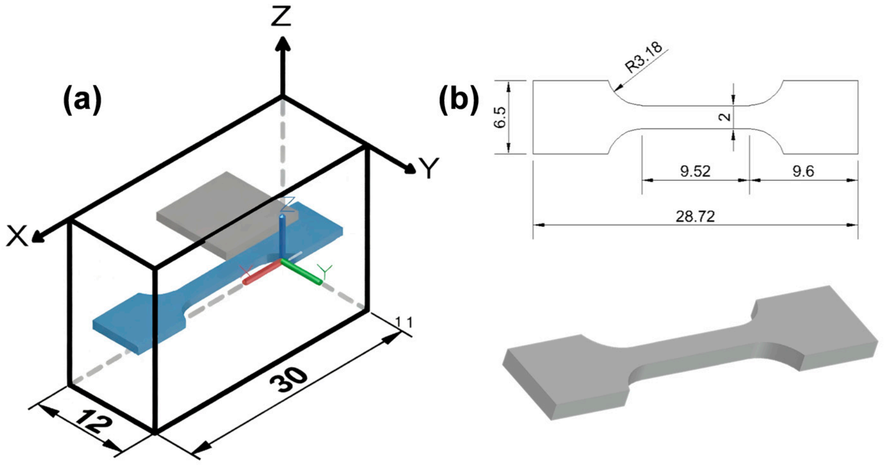

Supplementary Figure S2. SEM micrographs of the as-printed specimens and corresponding EDS mapping analysis results. The SEM micrographs demonstrate that the deposited samples are dense and homogeneous under the optimized process parameters. The EDS results show that with the increase in volumetric energy density, the distribution of Al and Fe elements gradually becomes more uniform at the microscale.

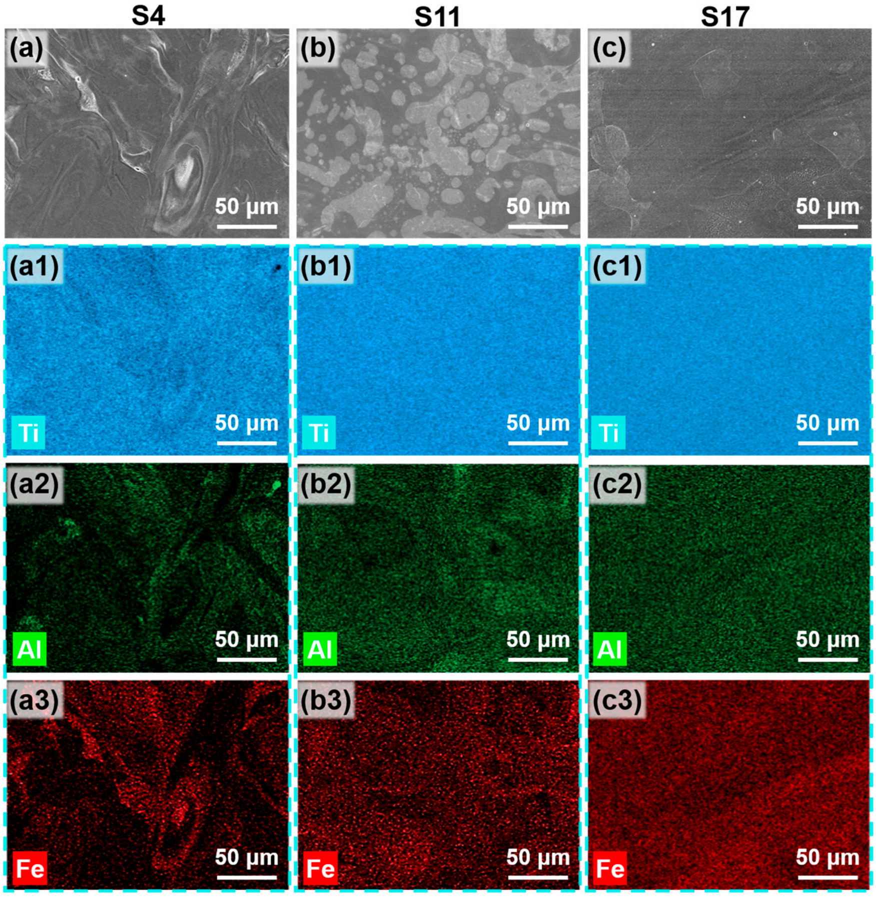

Supplement: Supplementary file 1 [file materials-18-04986-s001.zip › materials-3956960-supplementary.pdf]
